# Twin‐To‐Twin Transfusion Syndrome in Dichorionic‐Triamniotic Triplets Successfully Managed Without Fetoscopic Laser: A Management‐Focused Case Report

**DOI:** 10.1002/ccr3.73106

**Published:** 2026-07-06

**Authors:** Ammir Abuzahra, Safwat Jabari, Mohammad Rabaee, Saja Atawneh, Shaimaa Sweity, Rasmiya Qawasmi, Omar Hammam Salloum

**Affiliations:** ^1^ Department of Obstetrics & Gynecology, College of Medicine Hebron University Hebron Palestine; ^2^ Health Education and Scientific Research Unit Minstry of Health Ramallah Palestine; ^3^ Faculty of Medicine Palestine Polytechnic University Hebron Palestine

**Keywords:** cesarean section, low birth weight, neonatal intensive care, perinatal care, premature birth, triplet pregnancy, twin‐to‐twin transfusion syndrome

## Abstract

Triplet pregnancies are rare (~0.1% of births) and often complicated by prematurity. When one placental unit is monochorionic, there is a risk of twin‐to‐twin transfusion syndrome (TTTS), a severe vascular imbalance affecting monochorionic gestations. We report a 29‐year‐old primigravida with a dichorionic‐triamniotic triplet pregnancy complicated by Quintero stage II TTTS affecting the monochorionic twin pair. The patient was managed with close surveillance, serial amnioreductions, and antenatal corticosteroids. At 32 + 6 weeks' gestation, three male infants were delivered by cesarean section with favorable neonatal outcomes following neonatal intensive care. This case demonstrates that, in selected late‐gestation triplet pregnancies complicated by TTTS, conservative management with amnioreduction and planned preterm delivery may be a reasonable alternative when fetoscopic laser therapy is not feasible.

## Key Clinical Message

Twin‐to‐twin transfusion syndrome (TTTS) in triplet pregnancies requires individualized management based on gestational age, disease severity, technical feasibility, and available expertise. While fetoscopic laser surgery remains the preferred treatment when feasible, conservative management with close surveillance, amnioreduction, and optimized timing of delivery may result in favorable outcomes in selected late‐gestation cases.

## 1 Introduction

Triplet pregnancies are rare (~0.09% of deliveries), and associated with increased perinatal morbidity due to prematurity and growth complications [1]. Chorionicity significantly influences clinical outcomes, including fetal survival, neonatal morbidity, prematurity‐related complications, and long‐term neurodevelopmental outcomes. In dichorionic‐triamniotic triplets (i.e., one monochorionic diamniotic twin pair and a singleton), the monochorionic component is vulnerable to twin‐to‐twin transfusion syndrome (TTTS), a serious condition occurring in approximately 15% of monochorionic twin gestations [2]. In dichorionic triplet pregnancies, TTTS or early fetal loss has been reported in up to 30% of cases [3]. Although rare, TTTS in higher‐order multiples presents unique management challenges. The optimal management of TTTS in higher‐order multiple gestations remains unclear, particularly when diagnosed after 28 weeks where fetoscopic laser therapy may carry technical difficulty and uncertain benefit.

We present a case of stage II TTTS in a dichorionic‐triamniotic triplet pregnancy, managed conservatively with favorable neonatal outcomes. This report contributes novel clinical insight into the surveillance and perinatal care of high‐order multifetal gestations complicated by TTTS.

## 2 Case History / Examination

A 29‐year‐old primigravida (conceived with ovulation induction) was diagnosed with dichorionic‐triamniotic triplets consisting of a monochorionic diamniotic twin pair (Babies A and B) and a singleton (Baby C). The antenatal course was closely monitored. Maternal status remained stable throughout surveillance; she denied dyspnea, abdominal pain, or symptoms suggestive of mirror syndrome. Blood pressure remained within normal limits on serial assessments, urinalysis showed no proteinuria, and no clinical or laboratory features of preeclampsia developed. She reported intermittent uterine tightening without regular contractions and was managed expectantly as an outpatient.

Weekly ultrasound surveillance included fetal biometry, amniotic fluid assessment, umbilical artery Doppler, middle cerebral artery peak systolic velocity (MCA‐PSV), and ductus venosus Doppler evaluation of the monochorionic pair. Aside from abnormal umbilical artery Doppler in the donor twin, other Doppler parameters remained reassuring, and no hydrops fetalis was detected.

Between 28‐ and 30‐weeks' gestation, progressive discordance in amniotic fluid volumes developed within the monochorionic pair. The recipient twin (Baby A) demonstrated polyhydramnios with a maximum vertical pocket of approximately 10 cm, whereas the donor twin (Baby B) had severe oligohydramnios (amniotic fluid index approximately 2 cm) with a non‐visualized bladder. Umbilical artery Doppler assessment of the donor twin did not demonstrate persistent absent or reversed end‐diastolic flow. TTTS was classified using the Quintero staging system. These findings fulfilled the diagnostic criteria for Quintero stage II twin‐to‐twin transfusion syndrome, characterized by polyhydramnios–oligohydramnios sequence, non‐visualization of the donor bladder, and preserved Doppler studies. A multidisciplinary consultation involving maternal‐fetal medicine specialists and neonatologists was undertaken. Fetoscopic laser photocoagulation was considered; however, gestational age approaching the third trimester, uterine overdistension from triplet gestation, and the high likelihood of imminent preterm delivery were judged to limit expected procedural benefit while increasing intervention‐related risk. Therefore, a temporizing conservative strategy was chosen with the goal of prolonging gestation and improving neonatal maturity.

## 3 Methods (Differential Diagnosis, Investigations and Treatment)

Management consisted of serial amnioreduction of the recipient sac (approximately 1–1.5 L per session) and administration of antenatal corticosteroids (betamethasone) at 31 + 0 weeks.

At 32 + 6 weeks, the patient experienced spontaneous preterm labor. Due to fetal positioning (vertex–vertex–transverse lie), an emergency cesarean section was performed. Three male infants were delivered: Baby A (recipient twin) weighed 1950 g (Apgar scores 6 and 8 at 1 and 5 min), Baby B (donor twin) weighed 1400 g (Apgar scores 5 and 7), and Baby C (singleton) weighed 1770 g (Apgar scores 7 and 8). All three required initial stabilization in the delivery room, including stimulation and positive pressure ventilation as indicated. Baby B, the smallest, was intubated due to poor respiratory effort and received intratracheal surfactant for respiratory distress syndrome (RDS). Babies A and C were managed with continuous positive airway pressure (CPAP) for mild RDS. The placentas were examined following delivery. The monochorionic placental component demonstrated unequal placental sharing, consistent with the observed birth‐weight discordance between the monochorionic twins. These findings supported the coexistence of selective fetal growth restriction in the donor twin.

All three neonates were admitted to the neonatal intensive care unit (NICU) for thermoregulation, glucose monitoring, incubator care, and supportive respiratory and nutritional management. Baby B experienced transient hypoglycemia (blood glucose 38 mg/dL), which resolved with intravenous glucose; Babies A and C maintained normal glucose levels. Enteral feeds with expressed breast milk were initiated on Day 1 via orogastric tube and advanced cautiously. Baby B had mild feeding intolerance that was managed by slowing feed advancement; no necrotizing enterocolitis developed. Empiric intravenous antibiotics were administered for 48 h pending negative blood culture results. All infants required phototherapy for physiologic jaundice. Cranial ultrasonography was performed in all three neonates and demonstrated no evidence of intraventricular hemorrhage, periventricular leukomalacia, or other significant intracranial abnormalities.

By the end of the third week of life, all infants had transitioned to full oral feeds and were gaining weight appropriately. None developed bronchopulmonary dysplasia or other major complications. At discharge, Baby A and Baby C were discharged on Day 21 with weights of approximately 2260 g and 2100 g, respectively. Baby B was discharged on Day 32, at a corrected gestational age of 36 + 2 weeks (postnatal Day 32), weighing approximately 2000 g. All three neonates had stable vital signs, were feeding well, and demonstrated appropriate developmental progress for their corrected age at both discharge and at 3‐month follow‐up. The chronological sequence of clinical events is summarized in Table 1.

**TABLE 1:** Timeline of clinical course.

Gestational age	Event
28 weeks	Discordant amniotic fluid first detected
30 weeks	Stage II TTTS confirmed
31 + 0 weeks	Antenatal corticosteroids administered
31–32 weeks	Serial amnioreductions performed
32 + 6 weeks	Preterm labor → cesarean delivery
Birth	NICU admission for all neonates
Day 21–32	Gradual discharge of infants
3 months corrected age	Normal developmental follow‐up

## 4 Conclusions and Results (Outcome and Follow‐Up)

Twin‐to‐twin transfusion syndrome in dichorionic‐triamniotic triplet pregnancies presents unique management challenges. Although fetoscopic laser surgery remains the preferred treatment when technically feasible and performed by experienced fetal surgeons, management should be individualized according to gestational age, disease severity, anticipated interval to delivery, and procedural feasibility. In selected late‐gestation cases, conservative stabilization with amnioreduction, antenatal corticosteroids, close surveillance, and appropriately timed delivery may achieve favorable maternal and neonatal outcomes.

## 5 Discussion

Twin‐to‐twin transfusion syndrome (TTTS) is a significant complication unique to monochorionic gestations [2]. Its occurrence within a dichorionic‐triamniotic triplet pregnancy—where only one placental unit is shared—is uncommon but clinically important [4]. Early diagnosis through ultrasound surveillance is essential, as TTTS can progress rapidly and is associated with considerable perinatal morbidity and mortality [2].

In this case, classic Quintero stage II TTTS findings were present, including a marked polyhydramnios–oligohydramnios sequence, no visualization of the donor's bladder, with preserved Doppler studies. These features are consistent with the staging criteria described by Quintero et al. These features are consistent with the staging criteria described by Quintero et al. [5] They also help distinguish TTTS from selective fetal growth restriction, which does not typically present with such pronounced amniotic fluid discrepancy or the same Doppler changes [6].

Although fetoscopic laser photocoagulation is the recommended treatment for TTTS when feasible, with improved survival and reduced neurologic morbidity compared with conservative management [7]. Neonates affected by TTTS are often critically ill at birth and remain at substantial risk of mortality and severe morbidity. Lopriore et al. reported that TTTS pregnancies not treated with fetoscopic laser surgery had approximately threefold higher rates of neonatal mortality and/or severe morbidity compared with laser‐treated pregnancies. These findings highlight the severity of untreated TTTS and underscore the importance of both prenatal management and intensive neonatal care [8]. Current guidelines favor laser therapy before viability; however, after 28–30 weeks, the balance shifts toward stabilization and planned delivery. Our case supports this threshold in triplet pregnancies where procedural risk increases. Technical limitations in triplet pregnancies and the onset of preterm labor at 32 + 6 weeks in our patient prevented fetal intervention. In such situations, serial amnioreduction remains an acceptable alternative to temporarily stabilize hemodynamics and reduce uterine overdistension [6]. In this case, weekly amnioreductions, combined with antenatal corticosteroids and close fetal monitoring, contributed to the stability of both donor and recipient twins before delivery.

Outcomes for dichorionic–triamniotic triplet pregnancies complicated by TTTS vary. Chasen et al. documented that TTTS or fetal loss occurred in nearly one‐third of such pregnancies [3]. While Peeters et al. reported a perinatal survival rate of 76% in dichorionic triplets with TTTS, compared with only 51% in monochorionic triplets [4]. Additionally, donor twins typically face higher morbidity due to chronic hypovolemia, growth restriction, and metabolic instability [9]. The 28.2% birth‐weight discordance observed between the monochorionic twins also fulfilled criteria for selective fetal growth restriction, which may have contributed to the donor twin's increased neonatal vulnerability. In our case, the donor twin experienced transient hypoglycemia and respiratory distress but recovered without major complications—likely aided by early delivery, antenatal corticosteroids, and coordinated neonatal management.

A particularly notable aspect of this case is the excellent neonatal outcome for all three infants, including the donor twin, who was the most growth‐restricted. The donor's transient respiratory distress, hypoglycemia, and feeding immaturity are consistent with the expected physiologic vulnerabilities of TTTS donor twins [2]. Importantly, none of the infants developed serious complications such as bronchopulmonary dysplasia, necrotizing enterocolitis, severe intraventricular hemorrhage, or retinopathy of prematurity. Early transition to full oral feeds and appropriate weight gain further support the effectiveness of NICU management.

This case highlights several important clinical lessons: (1) TTTS must be considered in any triplet pregnancy that includes a monochorionic twin pair; (2) serial ultrasound surveillance is essential for timely identification of TTTS; (3) when fetoscopic laser therapy is not feasible, conservative management with amnioreduction and close monitoring may still result in acceptable outcomes; and (4) coordinated perinatal planning and NICU management play a critical role in optimizing survival and long‐term health in high‐order multifetal gestations.

Overall, this report adds useful clinical insight to the limited literature on TTTS in triplet pregnancies and demonstrates that favorable neonatal outcomes are achievable through vigilant surveillance and multidisciplinary care.

## Author Contributions

**Ammir Abuzahra:** conceptualization, project administration, supervision, writing – review and editing. **Safwat Jabari:** validation. **Mohammad Rabaee:** writing – original draft. **Shaimaa Sweity:** visualization. **Saja Atawneh:** data curation. **Rasmiya Qawasmi:** resources. **Omar Hammam Salloum:** supervision, writing – review and editing.

## Funding

The authors have nothing to report.

## Disclosure

CARE guidelines: This case report has been prepared in accordance with the CARE guidelines.

## Consent

Written informed consent was obtained from the patient for publication of this case report. A copy of the written consent is available for review by the Editor‐in‐Chief of this journal on request.

## Conflicts of Interest

The authors declare no conflicts of interest.

## Data Availability

Data available on request from the authors.
